# Clinical trial capacity building in a pandemic—outcome of a rapid site readiness project in Latin America

**DOI:** 10.3389/fpubh.2024.1179268

**Published:** 2024-04-25

**Authors:** Sue Ann Costa Clemens, Isabela Gonzalez, Daniele Sereni, Ralf Clemens

**Affiliations:** ^1^Institute for Global Health, University of Siena, Siena, Italy; ^2^Department of Pediatrics, Oxford University, Oxford, United Kingdom; ^3^Wit I.C.T. Consulting, Siena, Italy; ^4^International Vaccine Institute IVI BOT, Seoul, Republic of Korea

**Keywords:** site capacity building initiative, clinical trial site, COVID-19, Latin America, clinical development, success metrics

## Abstract

**Background:**

Latin America (Latam) has a tradition of large-scale vaccine trials. Because of fluctuating demand, many sites have downsized their infrastructure. Therefore, BMGF launched a clinical trial site-readiness initiative early in the coronavirus-2019 (COVID-19) pandemic including Latam countries between August and September 2020. This survey evaluated clinical development performance measures pre/post initiative (September 2022).

**Results:**

20/21 prequalified sites participated in COVID-19 vaccine/drug development trials. 156 clinical trials (140 COVID-19 vaccine/drug trials) were initiated in the 2 years since prequalification, compared to 176 in the 5 years before. 33,428/37,810 participants were included in COVID-19 programs. The number of enrolled subjects/day across sites quadrupled from 15 (1–35) to 63 (5–300). The dropout rate was 6.8%. Study approval timelines were reduced from 60 (12–120) to 35 (5–90) days. Mean qualified staff was increased from 24 (6–80) to 88 (22–180).

**Conclusion:**

Clinical trial sites across Latam were successfully prequalified to participate in COVID-19 developments. For the 100 days mission of vaccine availability in a new pandemic sufficient and well-trained clinical trial sites readily available are essential. This is only achievable if sites—especially in low/middle-income countries—are maintained active through a constant flow of vaccine studies.

## 1 Introduction

Timely vaccine development is paramount in pandemic management (1). Clinical trials are the gold standard for evidence generation, ensuring unbiased estimates of vaccine efficacy and safety data for vaccine emergency use authorization (EUA) and licensing. The coronavirus-2019 (COVID-19) pandemic exposed the need to improve the clinical trial research capacity in Low-middle-income countries (LMICs). Given the severe acute respiratory syndrome 2 (SARS-CoV-2) epidemiology, population diversity, and a long-standing tradition of large scale efficacy trials for global vaccine development [i.e., Cholera, Rotavirus, PCV, HPV, Dengue vaccines (2–6)], Latin America (Latam) was considered an attractive region for conducting vaccine trials during the SARS-CoV-2 pandemic. Unfortunately, because of fluctuating demand, many Latam sites engaged in previous large-scale trials had to downsize infrastructure, leading to an inability to engage quickly and efficiently at the beginning of the COVID-19 pandemic.

To support clinical trial site readiness and fast recruitment in the pandemic, the Bill & Melinda Gates Foundation (BMGF) launched, in August 2020, the COVID-19 site readiness initiative in Latam, Africa and Asia, to prepare sites and enhance their capabilities for conducting large scale COVID-19 vaccine trials. A grant was provided to the PDP partner “Instituto D’OR de Pesquisa e Ensino (IDOR),” as product development partner to prepare and qualify clinical trial sites for large scale COVID-19 vaccine trials in Latam. The project was implemented between August–November 2020 in 21 sites across 7 Latam countries (7). Sites which were qualified received grant funding to upgrade infrastructure, processes and resources for large trials in the midst of a pandemic. This paper discusses outcomes of the site readiness initiative.

## 2 Materials and methods

The aim of the BMGF project was clinical trial capacity building to ensure readiness for large scale COVID-19 vaccine development. Selection/qualification occurred between August–November 2020 and is described elsewhere (7). In short, potential sites were considered for inclusion if they had prior experience with vaccines or infectious disease clinical trials, were not yet involved in COVID-19 trials, could be qualified within 4 months including staff scale up, and were located in a country with EUA procedures in place. Of 34 potential sites from 10 countries, 22 sites in 7 countries were selected, trained and qualified. One site was qualified but, due to delays in regulatory site approval, was not included in the program. Qualified site information was shared on the COVAX website and directly with interested stakeholders.

Criteria of project success as defined by sponsor/principal investigator were:

1. ≥ 50% of qualified sites participated in COVID-19 vaccine development ≤4 months of qualification;
2. ≥1/4 of sites initiated their first COVID-19 study within 2020;
3. The number of trials at least doubled compared to the historical number;
4. Participant recruitment rate at least tripled compared to the historical number;
5. Dropout rate overall/by site of ≤10%;
6. Approval timelines, as an indirect measure of the site interaction with national regulatory authorities (NRAs) and institutional review boards (IRBs) ≤3 months;
7. Staff to meet trial demands (qualitative criterion).

To assess the outcomes of the site readiness initiative a follow-up questionnaire on key performance metrics was distributed by an external vendor in a Health Insurance Portability and Accountability Act (HIPAA) compliant online format to the principal investigators of the sites involved, in September 2022, 25 months after project initiation.

A descriptive analysis and comparison of clinical development metrics pre/post project based on the follow-up questionnaire was performed.

## 3 Results

Twenty of the qualified 21 sites (95%) across 7 countries were included in the COVID-19 vaccine development program: Brazil 6, Colombia 5, Dominican Republic 1, Guatemala 1, Honduras 1, Mexico 3, Peru 3 (Table 1). All sites had their first COVID-19 trial initiated within 4 months after qualification;6 started their first COVID-19 trials still in 2020, meeting the predetermined project goals 1 and 2.

**Table 1:** Sites qualified during the readiness project and involved in COVID-19 trials.

Country		City	Site name
1	Brazil	Porto Alegre	Hospital de Clinicas de Porto Alegre (HCPOA)
2	Brazil	Natal	Instituto Atena de Pesquisa Clinica
3	Brazil	Belem	Instituto Evandro Chagas
4	Brazil	Santa Maria	Universidade Federal de Santa Maria (UFSM)
5	Brazil	Rio de Janeiro	Instituto D’OR de Pesquisa e Ensino (IDOR Gloria D’OR)
6	Brazil	Natal	Centro de Estudos e Pesquisas em Molestias Infecciosas
7	Colombia	Barranquilla	Clinica de la Costa
8	Colombia	Bogota	Centro de Atencion e Investigacion Medica (Caimed)
9	Colombia	Cali	Centro de Estudios en Infectología Pediátrica (CEIP)
10	Colombia	Barranquilla	Clinica de la Costa
11	Colombia	Bogota	Centro de Estudios en Infectología Pediátrica (CEIP)
12	Dominican Republic	Santo Domingo	Fundacion Dominicana de Perinatología Pro-BEBE -HMNSA
13	Guatemala	Guatemala City	Centro de Estudos Clinicos Salud Avanzada (CECLISA)
14	Honduras	San Pedro Sula	Demedica
15	Mexico	Mexico City	Centro de Atención y Investigación Medica (Caimed)
16	Mexico	Guadalajara	CidVID Investigación Biomédica (IBIOMED)
17	Mexico	Aguascalientes	CidVID Investigación Biomédica (IBIOMED)
18	Peru	Lima	Investigaciones Medica en Salud
19	Peru	Lima	Instituto de Investigazion Nutricional
20	Peru	Lima	Universidad Peruana Caetano Heredia

Over the 5 years prior to the award, the 20 sites were executing 179 clinical trials (mean 5, range 0–12 per site) compared to 156 new clinical trials initiated in the 2-year survey period. Goal 3 was not met but the inclusion period was much shorter; 140/156 were COVID-19 trials. Trial sponsors were diverse—multinational companies, local/ global biotechs, academia and MoH’s as were vaccine platforms tested: mRNA, DNA, viral vector, inactivated, and adjuvanted protein subunit vaccines. Of note, 11/16 non-COVID-19 trials—mostly phase II/III—initiated during the pandemic were for other vaccines: chikungunya, RSV, dengue, norovirus, influenza and Ebola.

The main goals of the site readiness project were rapid high number recruitment into the COVID-19 clinical vaccine development and quality execution. The mean number of enrolled subjects/site/day by the 20 sites quadrupled from 15 (range 1–35) to 63 (range 5–300), meeting project goal 4. The total number of participants enrolled was 33,428 (6 sites ≥ 3,500 participants), mostly in COVID-19 vaccine efficacy trials. The abovementioned other vaccine programs included an additional 4,382 subjects, for a total number of 37,810 enrolled subjects in the approximately 25 months from qualification to survey.

Dropout rate, a key performance indicator of protocol adherence and study site quality, was on average 6.8%: 13 sites had dropout rates of ≤5%; 4 > 5%—≤10%, and 3 > 10%. The overall goal of dropout rate of less than 10% was met despite the complexity of doing clinical studies during a pandemic;17/20 sites also met the individual site goal.

A core element of the training process of sites was early engagement of local stakeholders (NRAs, IRBs) and involvement in study plans to reduce review/ approval timelines. This was achieved: timelines went down to 35 days (range 5–90) compared to the historical 60 days (range 12–120). Goal 6 was met, although it has to be acknowledged that the emergency procedures in place during the COVID-19 pandemic were most likely the main driver.

**Figure 1:**
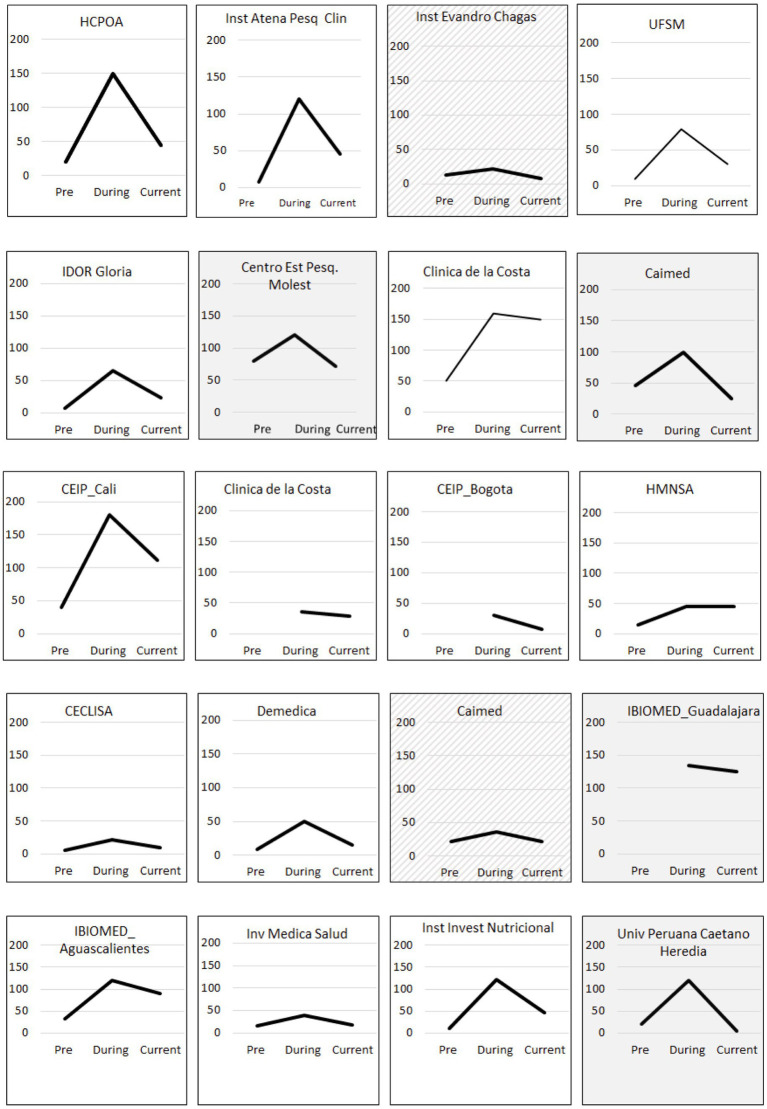
Total staff hired by site (y axis) before qualification (Pre), at the peak of demand during the COVID-19 pandemic (During) and by September 2022 (Current). Shaded and hashed boxes correspond to sites where current staff hired is lower or the same as before qualification, respectively.

The ability to have access to, hire and (re)train a sufficiently high number of staff in GCP/study procedures was critical for project success. Staffing increased from a mean of 24 across sites (range 6–80) to 88 (range 22–180) within 4 months (Figure 1).

## 4 Discussion

The COVID-19 fast vaccine development has substantially altered the course of the pandemic, saving millions of lives. A bottleneck in clinical development was the number of qualified trial sites able to initiate and execute large efficacy trials rapidly in the most affected regions. The BMGF clinical trial site readiness program was important in enabling Latam sites to fill that gap and help generate scientific evidence for rapid vaccine EUA.

In Latam, 20 clinical sites from 7 countries were qualified in the project. Details were immediately shared globally for suitable site selection. After site qualification, the number of participants/site/day quadrupled compared to historical data, regulatory/IRB approval timelines were reduced to half, and the number of trained staff was built up in time and scale to meet demand. As an example, four Brazilian sites that qualified early in the readiness project were successfully involved in conducting a large efficacy phase III trial recruiting, together with 2 other sites, 10,416 participants in 3 months. Participant retention rate and protocol compliance was high. Data generated contributed to about half of the clinical data used to approve the ChAdOx1 nCoV-19 Oxford AstraZeneca vaccine for emergency use globally (8, 9). Likewise, project sites in Colombia and Brazil contributed almost half of the 30,000 subject efficacy trial of an adjuvanted recombinant subunit vaccine developed by the Chinese biotech Clover and funded by CEPI, which now has EUA in China (10, 11).

The reduction of approval timelines is probably mainly due to changes in policies by- and international reliance between regulatory agencies and of IRBs. However, site staff was trained to interact with and involve agencies early in the projects to better understand their requirements upfront. Rapid participant recruitment and protocol adherence are critical for every successful clinical trial. Delays or quality issues in a trial lead to delays in EUA or licensure with a consequence of unnecessary morbidity and mortality. Therefore, the fast recruitment capability of the selected sites, with up to 300 participants enrolled per day, along with a low drop-out rate of just 1/15 participants in an ongoing pandemic reflect the success of the site readiness initiative. Comparisons of historical dropout rates are difficult as there are many confounders, such as trial duration, population, or number of invasive procedures. As the majority of participants were enrolled in efficacy trials with long-term follow-up, the benefit for the participant waned and adherence declined over the trial. National immunization policies changed during the study period. As participants were blinded, some opted to get COVID-19 vaccines from governmental sources. Another phenomenon contributing to drop-outs was vaccination tourism to the US due to easier vaccine access. Also, some participants did not adhere to the follow-up visits after being fully vaccinated to avoid social contact. However, from our own long experience with vaccine efficacy trials in Latam, a dropout rate of 6.8% in vaccine efficacy trials with long follow-up is reasonable. In another COVID-19 vaccine efficacy trial performed in Brazil and in sites which were not included in the site readiness project, the drop-out rate was more than double (12).

One of the cornerstones of quick recruitment is the training and qualification of new staff, which leads to high and fast participant enrolment, maintaining data quality and accuracy. Recruitment of staff was possible as there was a pool of individuals who had been involved in earlier projects but, because of instability in clinical trial demand, were made redundant. While recruitment was generally smooth, it still took substantial time to on-board the number of professionals needed to be up to the task. All re- or newly hired staff were highly motivated given the purpose for which they were hired. Our data show that most sites retained additional staff even after the peak in COVID-19 trials (during the pandemic) compared to before the readiness project (Figure 1).

An important lesson learned from this pandemic is that the retention of professionals in the regulatory and clinical trial environments in LMICs is key if we are to stand a chance to accomplish the ambition CEPI and governments to have vaccines available within 100 days of pathogen sequencing in a new pandemic. Clinical trial sites in LMICs, from where most likely novel pathogens will emerge, need to be kept “warm” by a constant flow of other vaccine studies in interpandemic times. This is a task and should also be a moral obligation for developing country vaccine manufacturers and even more so for the globally leading multinational companies (13).

There have been some positive trends recently with an increase in clinical trials including vaccines conducted in Latam. Data from clinicaltrials.gov shows that active vaccine trials in Latam went from 6 (2019–2020), to 16, 37 and 38 over the following 3 years. While encouraging, it is by far not enough to sustain infrastructures. The COVID-19 pandemic has underscored that the sustainability of clinical trial capacity in LMICs is crucial for the future, with increased focus on the preparedness for future epidemics and pandemics (14). As shown by our study, an improvement in clinical trial readiness has been achieved in Latam and needs to be maintained and cultivated, including the competitive timelines for regulatory and ethical approvals. Investments in local laboratory capacity building are mandatory, avoiding logistical challenges of shipping samples from Latam to labs in high income countries, which in the COVID-19 pandemic, with massive interruptions of air traffic, caused unnecessary delays. Finally, implementing a network of qualified sites with central/shared governance for attracting large-scale trials stands out as a priority.

Limitations of the global BMGF project were different speed of site qualification by geography, which was mainly based on the differences in prior site experience and hence baseline status. The site readiness was constantly provided to the COVAX website to which manufacturers had access. As more sites were qualified in Latam, and earlier, the jump in recruitment in Latam was just a pragmatic approach of developers and not an indication of a genuine preference over other geographies.

A limitation to our study is that the original project design, implementation and follow-up were carried out by the same team, which may have led to biases. However, performance assessment against preset metrics was done by an external vendor and shared with the project team. Another limitation is that the direct impact of the project on approval timelines is not quantifiable. Most likely, the EUA’s procedures were the main driver but the sites investigators facilitated the reviews and decisions by NRAs and IRBs through early and continuous interactions. Given site performance data is usually private and not publicly available, we could not adequately compare our outcome data with those of other sites outside our project. However, based on the experience of some authors, who have extensive large scale clinical development experience in the region, the project sites stand out with respect to time and quality metrics.

In conclusion, the BMGF site readiness project contributed to the successful inclusion of 20 qualified Latin American sites in COVID-19 clinical development, including vaccine efficacy trials, and thus providing scientific evidence for rapid vaccine EUA. This was important in the global context, and also regionally, by providing local data. The project was immediately accepted by potential sponsors as evidenced by the substantial increase in trials and subjects. High trial quality was maintained as evidenced by low dropout rates. For the 100 days ambition (14) for vaccine availability in a potential new pandemic, it is essential that sufficient and well-trained clinical trial sites are readily available. This can only be achieved if sites are maintained active not only in high income countries, but especially in LMICs through a constant flow of vaccine studies.

## Data availability statement

The raw data supporting the conclusions of this article will be made available by the authors, without undue reservation.

## Ethics statement

Ethical approval was not required for the study involving humans in accordance with the local legislation and institutional requirements. The studies were conducted in accordance with the local legislation and institutional requirements. Written informed consent to participate in this study was not required from the participants in accordance with the national legislation and the institutional requirements.

## Author contributions

SC, RC, and DS contributed to the conception and design of the study. DS organized the database. RC, DS, and IG wrote the first draft of the manuscript. All authors contributed to the article and approved the submitted version.
